# Traumatic Pseudoaneurysm of Superior Rectal Artery - an Unusual Cause of Massive Lower Gastrointestinal Bleed: A Case Report

**DOI:** 10.4021/gr274w

**Published:** 2011-01-20

**Authors:** Javid Iqbal, Lileswar Kaman, Mahesh Parkash

**Affiliations:** a Department of General Surgery, PGIMER, Chandigarh, India; b Department of Radio Diagnosis, PGIMER, Chandigarh, India

**Keywords:** Superior rectal artery, Traumatic pseudoaneurysm, Bleeding, Embolization

## Abstract

Traumatic pseudoaneurysm of superior rectal artery is an unusual cause of massive lower gastrointestinal bleed. We are reporting the first case as we could not come across any similar report in the literature. Patient underwent exploratory laparotomy, diversion sigmoid loop colostomy, perineal wound debridement and antiseptic dressing for traumatic perineal wound. Patient had repeated episode of massive lower gastrointestinal bleeding and was diagnosed as a case of bleeding from superior rectal artery pseudoaneurysm which was managed by selective superior rectal artery embolization after failure of surgical treatment.

## Introduction

The superior rectal artery is a terminal branch of inferior mesenteric artery. Pseudoaneurysm arising from superior rectal artery is a rare entity and selective embolization offers a viable option for treatment in patients presenting with massive lower gastrointestinal bleed [1]. Traumatic pseudoaneurysm of superior rectal artery has not been reported in literature. Here we are reporting a case of traumatic superior rectal artery pseudoaneurysm, who presented with repeated episodes of massive lower gastrointestinal bleed.

## Case Report

**Figure 1:**
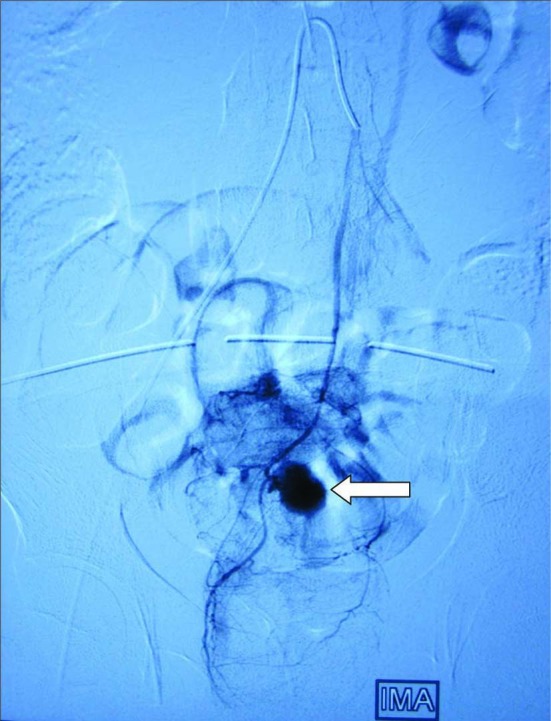
Angiogram showing pseudoanyreusm arising from superior rectal artery. White arrow pointing the pseudoanyreusm on angiogram.

**Figure 2:**
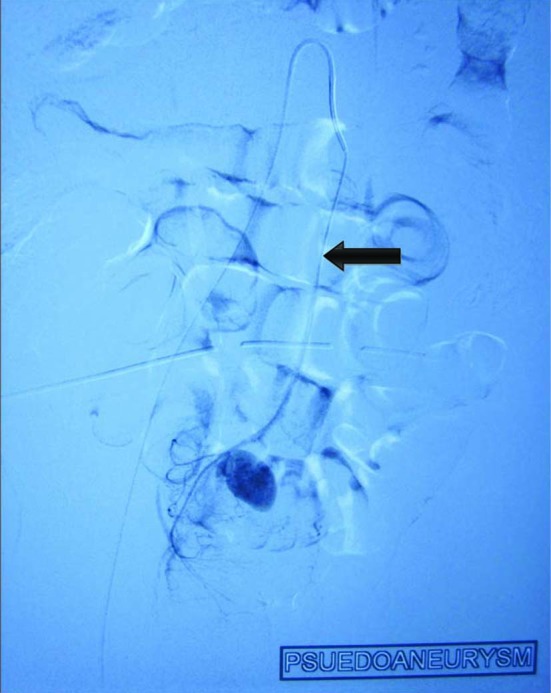
Showing microcather in situ and pseudoanyreusm arising from superior rectal artery. Black arrow showing microcather in superior rectal artery.

**Figure 3:**
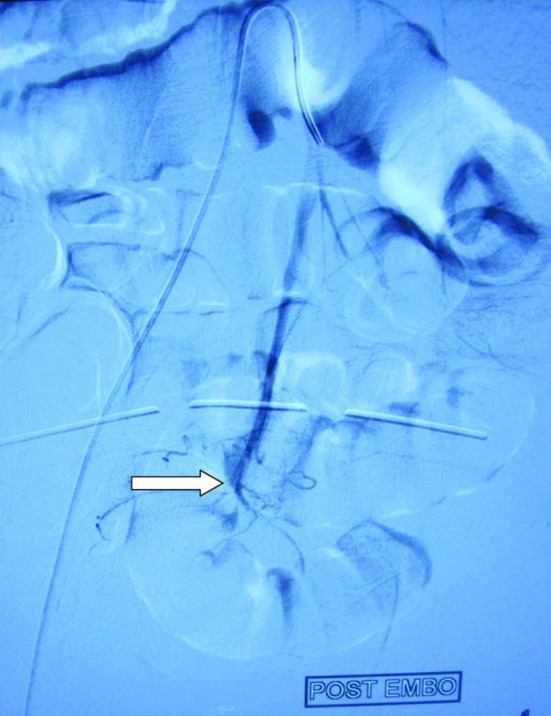
Showing no pseudoanyreusm after embolization. White arrow showing no pseudoanyreusm on check angiogram.

A 23-year-old male presented with a penetrating perineal wound which he sustained due to fall from height over an iron rod. Patient had bleeding from the perineal wound, which was managed at local hospital by wound debridement, packing and dressing. Patient started having bleeding from perineal wound and per rectum on the 11th day of trauma. Patient went into shock, for which he was resuscitated with crystalloid solution and 3 units of whole blood, packing of perineal wound and rectum done and was referred to tertiary health center. On reporting to our hospital, patient was conscious, oriented, febrile (38.7 *°*C) and pale. Pulse rate was 106/minute, BP: 90/54 mmHg, respiratory rate: 28/minute. Limbs, spine, chest, cardiovascular and abdominal examination were grossly normal. On local examination there was a 3 x 2 cm penetrating infected wound in perineal region on right side. On rectal examination sphincter tone was decreased and a rectal tear was present at 6 cm from the anal verge at 7 o’clock position with no active bleeding. Hemoglobin was 4.0 gm/dl, TLC: 33,000 and rest of blood analyses were normal. Wound swab showed growth of Enterococcus species, sensitive to amoxicillin. Blood culture showed growth of *E. coli*, sensitive to Imipenam and Cilastatin. Patient received 3 units of packed RBCs and intravenous antibiotics as per culture sensitivity. Patient underwent laparotomy, diversion sigmoid loop colostomy, perenial wound debridement and antiseptic dressing. Patient was febrile but haemodynamically stable in postoperative period. Patient started having massive bleeding per rectum on the second day of surgery. Hemostasis was achieved by rectal packing, which was removed after 24 hrs. Sigmoidoscopic examination revealed a rectal tear at 6 cm from the anal verge without any active bleed. On the 7th day of the surgery, patient had again massive lower gastrointestinal bleeding. Rectal packing was done on bedside after which blood started coming through stoma. After resuscitation, patient was taken for angiography and found to have 2 x 1.5 cm pseudoaneurysm (Fig. 1) arising from superior rectal artery for which selective superior rectal artery embolization was done with the help of microcathetar (Fig. 2) using 50%, 1 cc N-butyl cyanoacrylate (NBCA) mixed with lipoidal. Check angiogram showed no opacification of pseudoaneurysm (Fig. 3) and no procedure related complication noted. Bleeding stopped after embolization and recovered well. He was discharged with sigmoid loop colostomy in satisfactory condition and there was no recurrent bleeding during follow-up. After 6 weeks, sigmoid colostomy was reversed.

## Discussion

The vascular supply to the rectum is from a relatively rich anastomotic supply between branches of inferior mesentery artery and internal iliac arteries. The inferior mesenteric artery supplies the superior aspect of rectum through superior rectal artery and internal iliac arteries supply the lower one-half to two-thirds of the rectum through middle and inferior rectal arteries [1]. These arteries have an extensive anastomotic network within the wall of the rectum. The collateral blood allows a safety margin during embolization to prevent ischemia or infarction that can occur during colonic or small bowel embolization [2]. Nowadays, the technique of superselective embolisation offers a viable option for treatment of visceral bleed from vessels like superior and middle rectal artery [1, 3]. Angio-embolization via the superior rectal artery as a treatment modality for internal hemorrhoids and for lower gastrointestinal bleed in a case of multiple superior rectal artery microanyeursms has been reported in the literature [1, 4]. Traumatic superior rectal artery pseudoaneurysm as a cause of massive lower gastrointestinal bleed has not been reported in the literature. In our case, patient started having massive lower gastrointestinal bleeding which could not be controlled by repeated surgical attempts. Moreover, the access to the superior rectal artery was surgically difficult in this situation, and also the source of bleeding was not known during the surgical procedure. After the failure of second surgery to control the massive lower gastrointestinal bleeding, we started thinking about non-surgical modalities for the unusual source of bleeding. So the intervention radiologist was involved and patient was managed by selective embolization of superior rectal artery pseudoaneurysm by using NBCA mixed with lipoidal. NBCA, a liquid permanent embolic material, has been used in few case reports to treat arteriovenous malformation and life-threatening bleeding from the rectum and sigmoid colon [5-7]. Because of its low viscosity it can be injected via narrow-lumen catheters to embolize small arteries and collateral circulations, which are difficult to embolize with coils [8, 9]. However, to reduce the area at risk for ischemia, the most distal site should be chosen for embolization [1, 6]. Thus, superselective embolization of the superior rectal artery theoretically reduces the risk of ischemic complication of sigmoid colon and rectum. In our case, pseudoaneurysm arising from superior rectal artery was treated by embolization of superior rectal artery without any ischemic complication or rebleed. So, from our experience we conclude that in case of massive lower gastrointestinal bleeding after trauma early angiographic localization should be considered as a diagnostic and therapeutic modality.
